# Parentification in Polish Adolescents: a Prevalence Study

**DOI:** 10.1007/s40653-021-00411-8

**Published:** 2021-10-25

**Authors:** Judyta Borchet, Lisa M. Hooper, Sara Tomek, Wei S. Schneider, Maciej Dębski

**Affiliations:** 1 University of Gdańsk, Gdańsk, Poland; 2 University of Northern Iowa, Center for Educational Transformation, Cedar Falls, Iowa, USA; 3 Baylor University, Waco, TX USA

**Keywords:** Parentification, Prevalence, Adolescence, Poland

## Abstract

This study investigated the prevalence of parentification in a nationwide cross-sectional study. There were *N* = 47,984 Polish adolescents aged 12–21 (*M* = 15.60; *SD* = 1.98; female 52.7%, male 47.3%). The results indicated that more adolescents experienced emotional parentification (toward parents 35.9%; toward siblings 25.2%) as compared to instrumental parentification (toward parents 7.2%; toward siblings 15.5%), which is noteworthy, since emotional parentification is the most detrimental form of parentification in USA samples. Overall, 15.5% of the participants reported a sense of injustice related to their family caregiving roles and 61.2% reported satisfaction related to their family caregiving roles. The results are important given the dearth of prevalence studies.

Parentification is a potential form of maltreatment (Hooper, 2007; Jurkovic, 1997) and its manifestations may be characterized as emotional abuse, physical abuse, and neglect (Kerig, 2005; Nuttall et al., 2012). Similar to other forms of child maltreatment and neglect, the invisible impacts of parentification on childhood development and its short- and long-term consequences cannot be overstated (Hooper et al., 2009). Childhood is shaped by both parent relationships and family structures in which the child is embedded. Empirical evidence shows that childhood parentification thwarts healthy child development and growth across varied domains (educational, physical health, mental health, biological, and relational) and often foretells lifelong pernicious outcomes (Chen et al., 2018; Khafi et al., 2014). Meta-analytic evidence supports the link between childhood parentification and adult psychopathology (Hooper et al., 2011).

Parentification also is likely to be underreported and ubiquitous, since it remains unclear how prevalent parentification is in the US and globally. Currently, only one study (Siskowski, 2006) has reported on rates of childhood parentification (i.e., caregiving) in the United States, and no studies, of which we are aware, report on prevalence rates of parentification globally. As a preliminary step, we aimed to describe the prevalence rates of self-reported current levels of parentification in an understudied sample of Polish adolescents. The current study is derived from self-reports from a nationally representative sample of Polish adolescent participants.

## Background Literature

Parentification may be defined as the reversal of family roles and responsibilities between the parents and the children (Haxhe, 2016; Jurkovic, 1997). As a consequence, children and adolescents perform tasks and hold positions that are normatively assigned to adults in their culture (see Hooper, 2013). Such boundary dissolutions, family hierarchy distortions, and lack in parental care may stem from the intergenerational transmission of dysfunctional family roles and responsibilities (i.e., the parent’s own experience of childhood parentification and neglect), or be related to the parent’s inability to perform the parental role because of, for example, substance dependence, mental/physical illness, personality disorders, marital conflict, or migration and other issues (Hooper et al., 2011; Kerig, 2005; Nuttall et al., 2019). As a consequence, for children and adolescents, parentification may pose a threat to their development, impeding fulfillment of their developmental tasks, including learning at school or developing healthy peer relationships (Chase et al., 1998; Kerig, 2005; Siskowski, 2006).

Parentification may be classified by its type (dimension), the child’s care recipient/focus, and possible consequences (Hooper et al., 2011; Jurkovic, 1997). First, parentification can be labeled as emotional or instrumental. Emotional parentification refers to the child satisfying the emotional and social needs of other family members as well as facilitating a caring, positive atmosphere in the family, which may involve the child becoming, for example, a parental therapist or confidant (Hooper et al., 2011; Schier et al., 2015). Instrumental parentification relates to the involvement in functional tasks aimed at caring for the physical and daily living needs of the family members (e.g., cleaning, raising siblings, providing money for the family; Earley & Cushway, 2002; Hooper et al., 2011). Instrumental parentification is said to be less resource-consuming, less emotional-laden, less “abnormal,” and thus less detrimental to the child’s development as compared to emotional parentification (Byng-Hall, 2008; McMahon & Luthar, 2007; Tompkins, 2007). Yet, children may serve a variety of family roles, including both emotional and instrumental parentification occurring separately and /or simultaneously (Kerig, 2005; Khafi et al., 2014; Schier, 2014).

Second, parentification can be described based on to whom the child’s care is directed. Parent-focused parentification means the caregiving role and responsibilities are being directed toward the parent(s). Sibling-focused parentification means the caregiving role and responsibilities are being directed toward one’s sister, brother, or many siblings at the same time (Hooper et al., 2011). Importantly, those parentification dimensions are not mutually exclusive. The child may be burdened with responsibility and caregiving for parents, sibling(s), and/or other family members as well.

Third, parentification may be viewed in light of its consequences. A situation which goes beyond the child's capabilities (e.g., emotional and physical capacities) and exhausts resources that foretell numerous negative consequences such as anxiety, depression, personality disorders, and eating disorders (Arellano et al., 2018; Burton et al., 2018; Hooper et al., 2011), use of psychoactive substances (Chase et al., 1998; Dragan & Hardt, 2016), difficulties in relationships (Shaffer & Madden, 2016), poor academic performance (Burton, 2007; Chase et al., 1998; Siskowski, 2006), and poor parenting skills as an adult (Nuttall et al., 2019). In some circumstances, however, parentification may be beneficial. This experience may be accompanied with boosting one’s feeling of competence, self-esteem, resilience (Borchet et al., 2020b) and well-being (Burton et al., 2018), potentially lowering antisocial behavior (Chen et al., 2018), and facilitating positive relations with family members (Tompkins, 2007).

## Parentification as a Threat to Healthy Child Development and Outcomes

Parentified children are often assigned tasks and responsibilities that go beyond their developmental abilities, without receiving acknowledgment or adequate support from their caregivers or other family members. Age and developmental stage are factors related to the severity of outcomes reported among parentified youth (Burton et al., 2018; East, 2010; Khafi et al., 2014). The earlier in a child’s life parentification occurs, the more serious (e.g., adverse, traumatic, or long-lasting) are the consequences of parentification (Jurkovic, 1997; Schier, 2014). Young children typically are not resourceful enough to cope with caretaking assignments and behaviors compared to their older counterparts (e.g., adolescents or adults), irrespective of their pseudomaturity and competence they may present to the parent (Jurkovic, 1997) and others in the systems in which they are embedded (e.g., school or community). In fact, young children who are parentified are often characterized as mature minors given that on the surface these children may appear to be competent, organized, hardy, and resourceful (Chee et al., 2014; Garber, 2011; Hooper et al., 2009). Regardless of their mature presentation, parentified children and adolescents need to be identified and offered support as they are at risk of developing lifelong difficulties.

## Differential Effects of Outcomes and Sociodemographic Factors

In addition to the importance of age and developmental stage, other cultural and sociodemographic factors may be implicated in the prevalence rates of parentification. Those can be, for instance, the child’s or adolescent’s gender, family socioeconomic status, or the environmental context such as the inhabited city size. These sociodemographic factors may separately and taken together result in culturally-unique contexts that shape the effects and when and for whom parentification emerges (see Chee et al., 2014; McMahon & Luthar, 2007; Thomas, 2017). For example, factors such as gender and inhabited city size may have a unique effect on parentification in the Polish cultural setting, while other factors may be more culturally universal (e.g., age and family SES).

### Gender

The gender differences in parentification have been debated, given that findings are inconsistent. Studies that indicate there are no gender differences in parentification can be found (Cho & Lee, 2019; Vulliez‐Coady et al., 2013), as well as studies showing that females report higher parentification than males (Byng-Hall, 2008; Schier et al., 2015; Stein et al., 1999; Thomas, 2017) or that males report higher parentification than females do (Arellano et al., 2018; Hooper et al., 2015). At the same time, researchers point out that parentification in boys may be underreported and underestimated as they may be reluctant to admit to carrying out tasks that are considered as not in line with the gender stereotype. On the other hand, boys may be more willing to admit to performing tasks that are consistent with the notion of “traditional” gender roles (East, 2010; Khafi et al., 2014; McMahon & Luthar, 2007). It may be that gender differences in parentification emerge and change due to ongoing cultural changes (Jurkovic, 1997; Thomas, 2017). What aspects of parentification are culturally sanctioned may vary based on the cultural community (Hooper, 2013).

In Poland, there are two divergent cross-sectional investigations about the relation between gender and parentification among samples comprised of adolescents. One study indicated a small statistically significant association between being a girl and parentification (Żarczyńska-Hyla et al., 2019). The second study (Lewandowska-Walter et al., 2017) indicated that based on the gender, Polish adolescents may be prone to experience different types of parentification. Girls were more likely to experience emotional parentification, whereas boys tended to experience more instrumental parentification (Lewandowska-Walter et al., 2017).

### Family Socioeconomic Status (SES)

The relation between low SES and the level of parentification has been widely studied (e.g., Burton, 2007; Chee et al, 2014; McMahon & Luthar, 2007; Żarczyńska-Hyla et al., 2019). Not only prolonged financial disadvantage but also experiencing a sudden change in SES may be a risk factor or antecedent for parentification, for example, changes in SES during an economic crisis (Jelastopulu & Tzoumerka, 2013). Apart from having difficulties with providing for the family needs, low family SES can expose children to parentification in ways such as the absence of parents who work long hours, difficult housing conditions, as well as children and adolescents starting professional roles early, often at the expense of their education (Burton, 2007). Experiencing parentification in a financially struggling family does not have to be unequivocal. When children from economically disadvantaged families engage in prosocial behaviors that benefit the family and support their parents, at the same time they are agentic and being protective of their own interests (Chee et al., 2014).

Despite being given less scientific attention, economically privileged families have also been identified as a risk group of parentification. Children whose parents are highly devoted or “addicted” to work may be called upon performing various parental tasks (Carroll & Robinson, 2000). Winton (2003) implies that economically privileged parents may put on their children the emotional burden of meeting their high expectations to be successful and provide them pride. This role is congruent with the role of *the perfect child* or *the good child* (Boszormenyi-Nagy & Spark, 1973; Haxhe, 2016). In this role, the child is to bring the parents constant satisfaction and no worries while keeping her/his own requests and needs hidden (Haxhe, 2016). Winton (2003) conjectured that the role of the perfect child was associated with social class, and parents’ experiences (e.g., the child’s performance and competencies might be a part of social competition and an indicator of success for the middle-class parents). Thus, children who are pushed to satisfy their parents' expectations are experiencing emotional parentification (Winton, 2003).

As in the US, Polish literature recognizes family poverty as a risk factor of parentification (Schier, 2014). Emotional parentification was found to be correlated to lower financial well-being, yet the correlation was small (Żarczyńska-Hyla et al., 2019). Also, family SES was found to be positively correlated with the perceived benefits of parentification (Borchet, 2018). For example, children from higher-SES families may have access to better education and healthcare, and that may foster their development despite experiencing parentification (Borchet, 2018). The relation between the family SES and parentification may be non-linear and influenced by the type of parental roles performed by children and adolescents (Winton, 2003).

### Inhabited City Size

The ecological context influences human development (Bronfenbrenner, 1979). However, parentification studies rarely consider environmental factors such as inhabited city size or rural/urban area. In the US, one study investigated adolescent-parent dyads living in a rural community. Parentification had not only negative consequences, such as adolescent depressive symptoms, but it was also buffering the relation between parental alcohol use and adolescent’s depressive symptoms (Hooper et al., 2012). Nevertheless, a study investigating the relations between the inhabited area characteristics and the level of parentification was not found. The question asked in a study on parentification and parental alcohol misuse by Godsall et al. (2004, p. 800) ‘*Do environmental factors, such as urban, suburban, or rural status, buffer the effects of parental alcohol misuse on family functioning?*’ remains unanswered.

In the Polish cultural setting, a study by Żyrczyńska-Hyla et al. (2019) found no correlation between the level of parentification and place of residence considered bivariate (rural or urban). However, the bivariate consideration of the place of residence seems reductionist as there are many differences in Polish cities considering their size (e.g., in yearly income and poverty; Główny Urząd Statystyczny [Statistics Poland], 2019). Also, the differences between well-developed villages and less-developed cities may blur, and therefore the locality’s legal status (having the legal urban/rural status [pol. prawa miejskie]) may not be the best operationalization of place of residence. Thus, we decided on defining the city size as the number of inhabitants, considering it a more objective characteristic defining the locality. In summary, studying parentification in conjunction with the environmental context in which the family is embedded (e.g., city size) could provide additional insight into how ecological factors and resources shape the emergence of parentification.

In Poland, although the differences in living conditions between the cities and rural areas are decreasing since joining the European Union in 2004, the differences between city sizes are still noticeable and can be evidenced in personal yearly income and risk of poverty (Główny Urząd Statystyczny [Statistics Poland], 2019). In 2018, the average yearly income was definitely lower in rural areas (PLN 26,671) than in cities (PLN 34,293). There were also differences in its amount between 5 classes of cities by their size. Also, rural areas had the highest relative at-risk-of-poverty rate (20.8%), whereas for cities in total it was 10.8%, and cities’ results varied (Główny Urząd Statystyczny [Statistics Poland], 2019).

In summary, parentification is a complex and culturally embedded phenomenon that goes beyond family dysfunctions and is rooted in individual and family characteristics (e.g., child’s gender and age, family SES, family members’ health status), as well as in environmental factors. Therefore, while studying parentification among samples that are composed of individuals who are at high risk of experiencing parentification (e.g., children of substance-dependent parents or whose siblings suffer from a chronic condition), there is a need for investigating parentification among general population samples and from national and international communities.

## The Current Study

Parentification is known as a relatively common clinical phenomenon that affects many children and adolescents worldwide (Byng-Hall, 2008; Hooper et al., 2011). However, little is known about its actual prevalence as there is only one source assessing its prevalence in the US (see study on child caregiving by Siskowski, 2006). Surprisingly, there are no parentification prevalence studies published, not only in the Polish, but also in any other European cultural setting. Despite the growing scientific attention, the global literature devoted to parentification is limited. Even less is known about parentification in Poland, with only a single monograph on the subject written in the Polish language (Schier, 2014). A search for the phrase “Poland” and “parentification” in PsycINFO results in only nine (9) research articles (search date: 01–14-2021). Although there is one published study comprised of Polish adolescents describing sociodemographic factors related to parentification (Żarczyńska-Hyla et al., 2019), it is unclear how parentification was measured given the lack of detailed information provided. Until now, there has been no systematic investigation on the prevalence of parentification in a nationwide, broad, diverse, and representative sample of Polish adolescents.

The lack of empirical and rigorous research on the prevalence and incidence of parentification is a significant issue. Thus, the first objective of this study is to identify the prevalence of parentification in a nationally representative general sample of Polish adolescents. A second objective is to clarify the level of parentification based on sociodemographic variables (e.g., age, gender, family SES, and inhabited city size). We address two questions: (1) What is the current prevalence of parentification among Polish adolescents?; (2) How does parentification vary in the population as a function of sociodemographic variables such as age, gender, family SES, and the inhabited city size?

## Method

### Procedure

The project was conducted in January 2018 to June 2019. Recruitment of adolescents began by requesting access from local authorities who then submitted requests to local schools to participate in the study. For participating schools, after receiving parental consent, the survey was distributed to adolescents during school time in one of their classes. Responses were collected using the platform LimeSurvey on school computers with students working on separate computers at the same time. The survey included a total of 258 items (61 used in this study) and the students had 45 min to complete the survey. Responses were collected across all 16 voivodeships (main local government units) within Poland to obtain a sample representative of the students within the country. The numbers of participants by the voivodeships are presented in Appendix.

### Participants

The initial dataset contained responses from 50,135 adolescents participating in the project. Adolescents were between 12 to 21 years old and attending school within the country of Poland. A total of 2,152 (4.3%) participants were removed from the sample due to missing or incomplete data resulting in a final sample of 47,984 adolescents (M_age_ = 15.60; SD_age_ = 1.98). Girls constituted 72.7% (N = 25,290) and boys composed 47.3% (N = 22,694) of the sample. No information was collected related to race and ethnicity. Detailed demographic information for the sample of adolescents contained within the current study is contained in Table 1.

**Table 1:** Sample Demographic Characteristics (N = 47,984)

Demographic Measure	N	%
Age		
12	1271	2.6%
13	5896	12.3%
14	10,410	21.7%
15	7464	15.6%
16	5539	11.5%
17	8406	17.5%
18	4448	9.3%
19	3685	7.7%
20	800	1.7%
21	65	.1%
Gender		
Female	25,290	52.7%
Male	22,694	47.3%
School Type		
Elementary School	26,666	55.6%
Vocational School	1329	2.8%
Technical School	8091	16.9%
High School	8226	17.1%
Middle School	3295	6.9%
Other	377	.8%
Sibling Status		
Yes	40,661	84.7%
No	7323	15.3%
Family Socio-Economic Status		
0	265	.6%
1	105	.2%
2	299	.6%
3	1006	2.1%
4	2517	5.2%
5	10,256	21.4%
6	10,632	22.2%
7	12,255	25.5%
8	6909	14.4%
9	1994	4.2%
10	1746	3.6%
City size		
Above 250,000	8150	17.0%
From 100,000 to 249,999	6282	13.1%
From 50,000 to 99,999	5981	12.5%
From 25,000 to 49,999	6552	13.7%
From 10,000 to 24,999	9029	18.8%
From 5,000 to 9,999	3230	6.7%
Less than 5,000	8760	18.3%

Family Socio-Economic Status (SES), 0 is the lowest rung

### Measures

#### Demographic Items

Five demographic variables were included in the current study. Age was included as both a continuous variable and a variable grouped into three categories: early adolescence (12–14), middle adolescence (15–17), and late adolescence (18–21). Gender was considered bivariate (girl or boy). Family SES was measured using the MacArthur Scale of Subjective Social Status – Youth Version (Goodman et al., 2001). Response options for family SES used a Cantril ladder were ranging from 0 (poorest) to 10 (richest). City size was categorized as 7 different categories of the size of the city within which the adolescent lived: above 250,000, between 100,000 and 249,000, between 50,000 and 99,999, between 25,000 and 49,999, between 10,000 and 24,999, between 5,000 and 9,999, and less than 5,000. Participants were asked to report if they had siblings (bivariate as a yes/no variable). All demographic measures were treated as categorical measures. Frequencies of the demographic measures within the sample are included in Table 1.

#### Parentification Measure

Parentification was measured by the Parentification Questionnaire for Youth (PQY; Borchet et al., 2020a). The PQY contains 26 items, each measured using a 5-point Likert-type response scale ranging from 1 (Never true) to 5 (Always true). There are six total subscales in the PQY. Four of the subscales measured parentification for all participants (*N* = 47,984): Instrumental Parentification toward Parents (IPTP), Emotional Parentification toward Parents (EPTP), Satisfaction with Role (Played in the Family; SWR), and Sense of Injustice (SI). Two additional parentification subscales were used among the participants with siblings (*n* = 40,659): Instrumental Parentification toward Siblings (IPTS) and Emotional Parentification toward Siblings (EPTS). Each subscale score was calculated using the average score of the items contained within the subscale. Scores range from 1 to 5. Given the multi-dimensional nature of the measure, Borchet et al. (2020a) asserted that using a total score is not recommended.

##### Instrumental Parentification toward Parents

IPTP is defined as the financial aid and housework that the child may do to support their parents. This subscale was measured using four items. Sample items include, “I am asked to do the shopping more often than other members of the family” and “I work and contribute to the family budget.” The average across all participants was equal to 1.98 (*SD* = 0.77) with a median equal to 1.75.

##### Emotional Parentification toward Parents

EPTP is defined as taking care of the parent’s emotional condition/state. This subscale score was measured using four items. Sample items include, “I help my parent/parents make important decisions” and “I comfort my parents when they are sad.” The average across the entire sample of participants was equal to 2.86 (*SD* = 0.87) with a median equal to 2.75.

##### Satisfaction with Role (Played in the Family)

SWR is defined as the adolescent’s perception of their role within their family, for example, whether the adolescent feels appreciated or cooperates with their family. The SWR subscale was measured using four items. Sample items include, “I feel appreciated by my family” and “I have a feeling that our family is like a team and that we cooperate well.” The overall average SWR score was equal to 3.36 (*SD* = 0.91) with a median equal to 3.50.

##### Sense of Injustice

SI is defined as the feeling of being used, unseen, and/or underappreciated by their family members. This subscale was measured using five items. Sample items include, “I feel disappointed by my parents and other family members” and “Sometimes I think I’m more responsible than my parents are.” The average score for the SI across all adolescents was equal to 2.15 (*SD* = 0.95) with a median equal to 2.00.

##### Instrumental Parentification toward Siblings

The IPTS subscale is defined as instrumental care toward their siblings, which may include relieving siblings from the housework, helping them with school, and disciplining them. The IPTS subscale was measured using four items. Sample items include, “I am responsible for helping my siblings do their school homework” and “It’s mainly me who points out to my siblings that they behave rudely and brings them back to order.” The average score for all adolescents with siblings in the full sample was equal to 2.25 (*SD* = 0.92) with a median equal to 2.00.

##### Emotional Parentification toward Siblings

The EPS subscale is defined as caring for the emotional well-being of their siblings, such as comforting their siblings, worrying about them, and protecting siblings from their parents. The EPS subscale was measured using five items. Sample items include, “I worry about my siblings when I am not at home” and “I defend my siblings and explain them to my parents.” The average score for all adolescents who report having siblings within the full sample was equal to 2.51 (*SD* = 0.96) with a median equal to 2.40.

#### Data Cleaning

The process of data cleaning was comprised of three steps. The first two steps were initial data cleaning and were performed by the study’s principal investigator. First, the initial number of participants invited to the study was 61,164, although 21% of the initial sample did not complete the survey, mostly for technical problems with the Internet, and because they did not finalize the survey. Those observations were excluded, resulting in 50,135 observations left in the dataset. Second, due to a significant amount of missing data, additional data cleaning was executed. As a result, 49,254 observations remained in the dataset. Third, a thorough data cleaning based on the investigation of the response consistency and coding was performed. In this step, there were 2 co-investigators involved. From the 49,254 there were 1270 observations excluded, resulting in the final data analytic sample (*N* = 47,984).

#### Data Analytic Procedures

Average subscale scores for all six parentification measures (IPTP, EPTP, SWR, SI, IPTS, and EPTS) were calculated and compared across all four of the demographic measures (age group, gender, family SES, and city size). Differences were tested using two sets of MANOVAs, one set for the four measures using the full sample (IPTP, EPTP, SWR, SI) and one set using the two measures for the subsample of adolescents with siblings (IPTS, EPTS). Additionally, the mean values for the six parentification subscales were also trichotomized. The mean values were separated into no parentification reported (*M* = 1), some parentification reported (1 < *M* ≤ 3), and frequent parentification reported (*M* > 3). The number of adolescents falling into each of these categories was calculated and compared across the same four demographic measures. Differences were calculated using chi-squared contingency tables. Differences were tested using two sets of MANOVAs, one set for the four measures using the full sample (IPTP, EPTP, SWR, SI) and one set using the two measures for the subsample of adolescents with siblings (IPTS, EPTS). Effect sizes were computed using Eta-Squared, and post-hoc mean comparisons were conducted using Tukey’s multiple comparison procedure. All analyses were conducted using SPSS Version 26 (IBM Corp., Armonk, N.Y., USA).

## Results

### Mean Differences in Parentification

The overall mean parentification scores for the six subscales of parentification, IPTP, EPTP, SWR, SI, IPTS, and EPTS, across the entire sample, are reported in Table 2. In addition, mean differences were compared across a subset of demographic categories and are also reported in Table 2, with p-values for the test of mean differences and effect sizes reported in addition to the overall means separated by demographic category.

**Table 2:** Mean Differences and Effect Sizes in Parentification Subscales by Demographic Characteristics (n = 47,984)

Variable	Parentification dimensions					
	IPTP	EPTP	SWR	SI	IPTS*	EPTS*
Overall Sample	1.984	2.857	3.357	2.153	2.247	2.513
Age Group						
Early Adolescence (12–14)	1.995	2.876	3.470	2.071	2.218	2.527
Middle Adolescence (15–17)	1.931	2.835	3.320	2.181	2.253	2.502
Late Adolescence (18–21)	2.090	2.870	3.223	2.243	2.290	2.514
F-test	*p* < .001	*p* < .001	*p* < .001	*p* < .001	*p* < .001	*p* = .065
Eta Squared	.006	< .001	.010	.005	.001	< .001
Gender						
Female	1.830	2.888	3.326	2.141	2.231	2.578
Male	2.156	2.822	3.392	2.166	2.265	2.440
F-test	*p* < .001	*p* < .001	*p* < .001	*p* = .004	*p* < .001	*p* < .001
Eta Squared	.045	.001	.001	< .001	< .001	.005
Family SES						
0	2.141	2.379	2.518	2.352	2.089	2.107
1	2.060	2.652	2.645	2.400	2.324	2.530
2	2.183	2.673	2.605	2.825	2.387	2.517
3	2.034	2.658	2.692	2.672	2.243	2.353
4	1.972	2.658	2.686	2.516	2.183	2.385
5	1.996	2.789	3.171	2.263	2.237	2.472
6	1.944	2.826	3.344	2.112	2.219	2.473
7	1.940	2.888	3.485	2.033	2.227	2.521
8	1.950	2.974	3.635	1.970	2.247	2.585
9	2.031	3.031	3.695	2.019	2.336	2.687
10	2.479	3.081	3.528	2.481	2.627	2.818
F-test	*p* < .001	*p* < .001	*p* < .001	*p* < .001	*p* < .001	*p* < .001
Eta Squared	.018	.015	.071	.035	.007	.009
City Size						
Above 250,000	1.934	2.837	3.321	2.152	2.196	2.445
From 100,000 to 249,999	1.961	2.856	3.368	2.153	2.208	2.492
From 50,000 to 99,999	1.945	2.818	3.273	2.171	2.217	2.463
From 25,000 to 49,999	1.994	2.855	3.321	2.176	2.268	2.537
From 10,000 to 24,999	2.002	2.866	3.348	2.162	2.261	2.536
From 5,000 to 9,999	2.021	2.860	3.395	2.147	2.299	2.565
Less than 5,000	2.036	2.893	3.462	2.115	2.286	2.555
F-test	*p* < .001	*p* < .001	*p* < .001	*p* = .002	*p* < .001	*p* < .001
Eta Squared	.002	.001	.004	< .001	.002	.002

*IPTP* Instrumental parentification toward parents, *EPTP* Emotional parentification toward parents, *SWR* Satisfaction with the role, *SI* Sense of injustice, *IPTS* Instrumental parentification toward siblings, *EPTS* Emotional parentification toward siblings; Family SES, 0 is the lowest rung

^*^limited sample: *n* = 40,659 adolescents with siblings

#### Age Group Differences in Parentification

The multivariate test for significant mean differences between the three age group categories (early adolescence, middle adolescence, and late adolescence) was significant for the IPTP, EPTP, SWR, and SI subscales, Λ = .979, *F*(8, 95,956) = 131.05, *p* < .001, as well as the IPTS and EPTS subscales for adolescents with siblings, Λ = .998, *F*(4, 81,310) = 17.05, *p* < .001. Significant mean differences were found across all three groups for the IPTP, *F*(2, 47,980) = 140.55, *p* < .001, the SWR, *F*(2, 47,980) = 253.99, *p* < .001, and the SI, *F*(2, 47,980) = 115.32, *p* < .001, subscales. For the IPTP subscale, early adolescence (12–14) had the lowest mean, middle adolescence (15–17) had a significantly larger mean, and older adolescence (18–21) reported the highest mean IPTP. For the SWR subscale, early adolescence had the highest mean, with middle adolescence reporting a significantly lower mean, and older adolescence reporting the lowest mean SWR scores. For the SI subscale, early adolescence reported the lowest mean, with middle adolescence reporting a significantly higher mean, and older adolescence reporting the highest mean SI scores. Significant mean differences were also found for the EPTP subscale, *F*(2, 47,980) = 11.87, *p* < .001, with middle adolescence scoring significantly lower than adolescents in either the early or older adolescence group, who did not significantly differ from each other. Significant mean differences were also found for the IPTS subscale across the three age groups, *F*(2, 40,656) = 16.21, *p* < .001, with early adolescence reporting the lowest mean, middle adolescence reporting a significantly higher mean, and later adolescence reporting the highest average IPTS subscale score. There were no significant differences between the three age groups on the average EPTS subscale scores, *F*(2, 40,656) = 2.74, *p* = .065.

#### Gender Differences in Parentification

The multivariate test for gender differences was found to be significant for the IPTP, EPTP, SWR, and SI subscale scores, Λ = .929, *F*(4, 47,978) = 918.52, *p* < .001, as well as the IPTS and EPTS subscales for adolescents with siblings, Λ = .990, *F*(2, 40,656) = 209.07, *p* < .001. Females had significantly higher mean scores for EPTP, *F*(1, 47,981) = 70.30, *p* < .001, and EPTS, *F*(1, 40,657) = 209.27, *p* < .001. In contrast, males had significantly higher mean scores on IPTP, *F*(1, 47,981) = 2266.81, *p* < .001, SWR, *F*(1, 47,981) = 62.77, *p* < .001, SI, *F*(1, 47,981) = 8.36, *p* < .001, and IPTS, *F*(1, 40,657) = 13.78, *p* < .001.

#### Family SES Differences in Parentification

The multivariate test for the mean differences between adolescents with different family SES was found to be significant for the IPTP, EPTP, SWR, and SI subscale scores, Λ = .901, *F*(4, 181,894.7) = 126.27, *p* < .001, as well as for the IPTS and EPTS subscales for adolescents, Λ = .987, *F*(20, 81,294) = 26.28, *p* < .001. Mean scores significantly differed for adolescents with different family SES values for the IPTP subscale, *F*(10, 47,972) = 87.63, *p* < .001, the EPTP subscale, *F*(10, 47,972) = 70.76, *p* < .001, the SWR subscale, *F*(10, 47,972) = 367.16, *p* < .001, and the SI subscale, *F*(10, 47,972) = 174.38, *p* < .001. Significant differences between family SES values were also found on the IPTP subscale, *F*(10, 40,648) = 29.66, *p* < .001, and the EPTS subscales, *F*(10, 40,648) = 36.21, *p* < .001, for adolescents with siblings.

In relation to the IPTP subscale, adolescents viewing themselves in the highest SES grouping (10), had the highest mean IPTP, with this value significantly higher than every other family SES “ladder rung” category. Those adolescents indicating family SES values of 4, 6, 7, and 8 had the lowest IPTP scores. Aside from the exception of the highest SES grouping, in general, adolescents with lower family SES scores had higher average IPTP scores. In relation to EPTP, there was an almost perfect positive relationship between EPTP scores and family SES. Adolescents indicating the lowest SES “ladder rung” had the lowest EPTP subscale score, and adolescents with the highest family SES “ladder rung” had the highest average EPTP subscale score, with significant mean differences between the lower categories (0–5) and the higher categories (6–10), and mean differences as you moved further up the family SES scale.

With relation to the SWR subscale, a similar positive relationship was also found. Adolescents indicating the lowest three family SES “ladder rung” categories had the lowest mean SWR subscale scores, and adolescents indicating the highest three family SES categories having the highest mean SWR subscale scores. With relation to the SI subscale scores, there was a general negative relation between family SES and SI subscale scores, with a few exceptions. The lowest mean SI subscale scores were those adolescents indicating the SES categories of 6, 7, 8, and 9, while the highest three mean SI scores were for the categories 2, 3, and 4. Both the highest and the lowest family SES categories were similar to each other and fell in the middle of the distribution of mean SI subscale scores, which is different from what one would expect with a negative relationship.

For adolescents with siblings, the distributions for both the IPTS and EPTS subscales were similar but did not present any clear patterns in terms of the relationship between family SES scores and the IPTS and EPTS subscales. For both the IPTS and EPTS subscales, the lowest mean values were for those adolescents indicating the lowest family SES category and the highest mean values were for those adolescents indicating the highest family SES category. In between those two categories, however, there does not appear to be a clear pattern of mean differences to infer a patterned relationship between family SES and either the IPTS or EPTS subscale scores.

#### City Size Differences in Parentification

The multivariate test for the mean differences between city size in which the adolescent resides and their IPTP, EPTP, SWR, and SI subscale scores was found to be significant, Λ = .994, *F*(24, 167,359.01) = 12.89, *p* < .001, as well as the IPTS and EPTS subscales for adolescent with siblings, Λ = .998, *F*(12, 81,302) = 7.98, *p* < .001. Univariate mean differences were found for each of the six subscales based on city size, IPTP, *F*(6, 47,976) = 18.25, *p* < .001, EPTP, *F*(6, 47,976) = 5.45, *p* < .001, SWR, *F*(6, 47,976) = 32.99, *p* < .001, SI, *F*(6, 47,976) = 3.46, *p* = .002, IPTS, *F*(6, 40,652) = 10.36, *p* < .001, and EPTS, *F*(6, 40,652) = 13.25, *p* < .001, subscale scores. In relation to the IPTP subscale, there was a clear negative relationship between mean IPTP scores and city size. The largest mean value was found for the smallest city size and the lowest mean value was found for the largest city size, with the city sizes in between falling in between these two values. In relation to the EPTP and SWR subscales, the negative relation was present, but not as clear with regards to the patterns of the mean values as compared to the IPTP subscale.

The highest EPTP and SWR subscale scores were found for the smallest city size, but the lowest mean was found for the adolescents living in cities sized 50,000 to 99,999, though this mean did not significantly differ from the largest city size, which was slightly higher for both subscales. Most city sizes within the middle of the range of city sizes did not significantly differ in terms of the EPTP and SWR subscales scores. City size did not present a clear relation with regards to mean SI values. The lowest mean SI score was found for adolescents in the smallest cities; however, these did not significantly differ from the largest cities. The largest mean SI scores were found in the mid-range cities. With regards to adolescents with siblings, both the IPTS and EPTS scores present a clear negative relation between mean scores and the city sizes. The largest sized cities have the lowest mean IPTP and EPTS subscale scores, while the smallest sized cities have the highest mean IPTS and EPTS subscale scores. Mid-sized cities fall between these two groups, with significant differences between the large and the small-sized cities.

### Response Prevalence in Parentification

In order to provide clarity related to the context to the scale responses and the significant differences between the demographic categories, mean response options for all six parentification subscale scores were trichotomized into the following categories: no parentification present (*M* = 1), low parentification present (1 < *M* ≤ 3), and high parentification present (*M* > 3). This analysis will allow for us to determine response prevalence of options, which will provide context for how adolescents were responding when mean differences were found for the demographic variables. Overall response rates, along with demographic differences in the response options, are displayed in Table 3.

**Table 3:** Differences in Response Percentages for Parentification Subscales by Demographic Characteristics (n = 47,984)

Variable	Parentification Dimensions by Score Category																	
	IPTP			EPTP			SWR			SI			IPTS*			EPTS*		
	*M* = 1	1 < *M* ≤ 3	*M* > 3	*M* = 1	1 < *M* ≤ 3	*M* > 3	*M* = 1	1 < *M* ≤ 3	*M* > 3	*M* = 1	1 < *M* ≤ 3	*M* > 3	*M* = 1	1 < *M* ≤ 3	*M* > 3	*M* = 1	1 < *M* ≤ 3	*M* > 3
Overall Sample	9.3% (4440)	83.5% (40,077)	7.2% (3467)	2.2% (1047)	61.9% (29,710)	35.9% (17,227)	1.5% (711)	37.3% (17,922)	61.2% (29,351)	14.0% (6725)	70.5 (33,811)	15.5% (7447)	11.4% (4616)	73.1% (29,727)	15.5% (6316)	7.3% (2959)	67.5% (27,455)	25.2% (10,245)
Age Group																		
Early Adolescence (12–14)	9.0% (1582)	83.5% (14,674)	7.5% (1321)	2.0% (345)	61.2% (10,761)	36.8% (6471)	1.3% (222)	32.3% (5685)	66.4% (11,670)	15.7% (2766)	70.7% (12,435)	13.5% (n = 2375)	11.5% (1690)	73.6% (10,823)	14.9% (2188)	6.6% (972)	67.6% (9942)	25.8% (3787)
Middle Adolescence (15–17)	10.1% (2171)	83.6% (17,899)	6.3% (1339)	2.2% (478)	62.8% (13,445)	35.0% (7486)	1.5% (321)	39.0% (8352)	59.5% (12,736)	13.1% (2806)	70.6% (15,122)	16.3% (3481)	11.1% (2015)	73.0% (13,283)	16.0% (2905)	7.3% (1334)	67.8% (12,347)	24.8% (4522)
Late Adolescence (18–21)	7.6% (687)	83.4% (7504)	9.0% (807)	2.5% (224)	61.2% (5504)	36.3% (3270)	1.9% (168)	43.2% (3885)	55.0% (4945)	12.8% (1153)	69.5% (6254)	17.7% (1591)	11.7% (911)	72.5% (5621)	15.8% (1223)	8.4% (653)	66.6% (5166)	25.0% (1936)
χ^2^	*p* < .001	*p* = .923	*p* < .001	*p* = .020	*p* = .074	*p* = .008	*p* = .001	*p* < .001	*p* < .001	*p* < .001	*p* = .356	*p* < .001	*p* = .236	*p* = .750	*p* = .035	*p* < .001	*p* = .715	*p* = .258
Gender																		
Female	11% (2772)	85.0% (21,498)	4.0% (1020)	1.8% (465)	59.8% (15,126)	38.4% (9699)	1.4% (349)	38.6% (9766)	60.0% (15,175)	14.4% (3652)	69.3% (17,528)	16.3% (4110)	10.4% (2231)	74.0% (15,928)	15.6% (3367)	5.7% (1222)	66.0% (14,201)	28.4% (6103)
Male	7.3% (1668)	81.9% (18,579)	10.8% (2447)	2.6% (582)	64.3% (14,584)	33.2% (7528)	1.6% (362)	35.9% (8156)	62.5% (14,176)	13.5% (3073)	71.8% (16,283)	14.7% (3337)	12.5% (2385)	72.1% (13,799)	15.4% (2949)	9.1% (1737)	69.3% (13,254)	21.6% (4142)
χ^2^	*p* < .001	*p* < .001	*p* < .001	*p* < .001	*p* < .001	*p* < .001	*p* = .054	*p* < .001	*p* = .001	*p* = .008	*p* = .002	*p* < .001	*p* < .001	*p* = .019	*p* = .515	*p* < .001	*p* < .001	*p* < .001
Family SES																		
0	29.8% (79)	52.8% (140)	17.4% (46)	29.4% (78)	44.9% (119)	25.7% (68)	27.2% (72)	41.5% (110)	31.3% (83)	30.2% (80)	46.4% (123)	23.4% (62)	35.5% (72)	48.8% (99)	15.8% (32)	35.5% (72)	48.8% (99)	15.8% (32)
1	17.1% (18)	72.4% (76)	10.5% (11)	11.4% (12)	54.3% (57)	34.3% (36)	11.4% (12)	52.4% (55)	36.2% (38)	14.3% (15)	59.0% (62)	26.7% (28)	17.0% (15)	64.8% (57)	18.2% (16)	10.2% (9)	69.3% (61)	20.5% (18)
2	9.7% (29)	78.3% (234)	12.0% (36)	6.7% (20)	62.5% (187)	30.8% (92)	6.0% (18)	65.9% (197)	28.1% (84)	6.7% (20)	55.5% (166)	37.8% (113)	12.3% (31)	64.3% (162)	23.4% (59)	13.1% (33)	58.7% (148)	28.2% (71)
3	8.6% (87)	84.0% (845)	7.4% (74)	3.7% (37)	67.2% (676)	29.1% (293)	3.6% (36)	67.4% (678)	29.0% (292)	6.3% (63)	62.1% (625)	31.6% (318)	14.5% (123)	68.6% (584)	16.9% (144)	12.5% (106)	66.9% (569)	20.7% (176)
4	9.1% (230)	85.3% (2148)	5.5% (139)	3.2% (80)	68.5% (1725)	28.3% (712)	2.4% (60)	59.6% (1500)	38.0% (957)	6.1% (153)	66.8% (1681)	27.1% (683)	12.6% (269)	74.3% (1585)	13.1% (279)	9.7% (206)	67.5% (1440)	22.8% (487)
5	8.5% (870)	85.1% (8729)	6.4% (657)	2.4% (244)	64.9% (6656)	32.7% (3356)	1.5% (151)	46.6% (4778)	51.9% (5327)	11.0% (1132)	71.2% (7307)	17.7% (1817)	11.4% (1007)	73.9% (6501)	14.7% (1291)	7.6% (673)	68.8% (6056)	23.5% (2070)
6	8.5% (908)	85.9% (9135)	5.5% (589)	1.6% (168)	64.1% (6817)	34.3% (3647)	0.8% (82)	38.3% (4072)	60.9% (6478)	12.1% (1285)	74.7% (7937)	13.3% (1410)	10.6% (957)	74.9% (6734)	14.5% (1300)	6.6% (597)	70.3% (6323)	23.0% (2071)
7	9.2% (1125)	84.7% (10,380)	6.1% (750)	1.5% (187)	61.5% (7542)	36.9% (4526)	0.9% (113)	30.8% (3774)	68.3% (8368)	15.5% (1899)	72.3% (8865)	12.2% (1491)	10.4% (1083)	74.9% (7788)	14.7% (1526)	5.9% (609)	69.1% (7189)	25.0% (2599)
8	9.7% (668)	82.9% (5726)	7.5% (515)	1.3% (87)	57.5% (3975)	41.2% (2847)	0.8% (57)	25.1% (1731)	74.1% (5121)	19.6% (1354)	68.7% (4746)	11.7% (809)	11.5% (674)	72.4% (4242)	16.1% (946)	6.5% (382)	65.2% (3823)	28.3% (1657)
9	10.0% (199)	79.6% (1588)	10.4% (207)	1.5% (29)	54.5% (1086)	44.1% (879)	1.0% (20)	24.1% (481)	74.9% (1493)	19.4% (386)	67.7% (1349)	13.0% (259)	11.0% (183)	70.0% (1168)	19.0% (318)	6.4% (106)	60.6% (1012)	33.0% (551)
10	13.0% (227)	61.6% (1076)	25.4% (443)	6.0% (105)	49.8% (870)	44.2% (771)	5.2% (90)	31.3% (546)	63.6% (1110)	19.4% (338)	54.4% (950)	26.2% (457)	14.3% (202)	57.1% (807)	28.6% (405)	11.7% (166)	52.0% (735)	36.3% (513)
χ^2^	*p* < .001	*p* < .001	*p* < .001	*p* < .001	*p* < .001	*p* < .001	*p* < .001	*p* < .001	*p* < .001	*p* < .001	*p* < .001	*p* < .001	*p* < .001	*p* < .001	*p* < .001	*p* < .001	*p* < .001	*p* < .001
City Size																		
Above 250,000	9.8% (800)	84.1% (6856)	6.1% (494)	2.4% (192)	63.1% (5146)	34.5% (2812)	1.4% (118)	39.1% (3187)	59.4% (4845)	13.3% (1084)	71.1% (5795)	15.6% (1271)	12.5% (806)	72.8% (4675)	14.7% (944)	8.4% (542)	68.6% (4405)	23.0% (1478)
From 100,000 to 249,999	9.5% (597)	83.8% (5264)	6.7% (421)	2.4% (149)	61.5% (3864)	36.1% (2269)	1.3% (80)	37.1% (2333)	61.6% (3869)	14.2% (889)	70.5% (4430)	15.3% (963)	12.1% (609)	73.8% (3726)	14.2% (717)	7.9% (397)	67.7% (3419)	24.5% (1236)
From 50,000 to 99,999	10.0% (598)	83.6% (5002)	6.4% (381)	2.5% (148)	62.3% (3729)	35.2% (2104)	1.7% (101)	40.8% (2443)	57.5% (3437)	13.5% (809)	70.4% (4213)	16.0% (959)	13.7% (678)	70.0% (3456)	16.2% (802)	8.2% (406)	67.4% (3325)	24.4% (1205)
From 25,000 to 49,999	9.3% (610)	83.3% (5457)	7.4% (485)	2.1% (139)	61.8% (4052)	36.0% (2361)	1.6% (107)	38.7% (2533)	59.7% (3912)	13.5% (882)	70.3% (4608)	16.2% (1062)	11.0% (620)	73.6% (4152)	15.4% (866)	7.3% (413)	67.1% (3785)	25.5% (1440)
From 10,000 to 24,999	8.8% (792)	83.7% (7558)	7.5% (679)	2.1% (193)	61.6% (5566)	36.2% (3270)	1.4% (124)	37.4% (3375)	61.2% (5530)	14.1% (1274)	70.2% (6342)	15.6% (1413)	10.8% (852)	73.4% (5765)	15.7% (1237)	6.9% (543)	67.1% (5270)	26.0% (2041)
From 5,000 to 9,999	8.3% (268)	84.3% (2722)	7.4% (240)	2.0% (64)	62.2% (2009)	35.8% (1157)	1.3% (42)	36.5% (1178)	62.2% (2010)	13.2% (426)	72.0% (2325)	14.8% (478)	9.6% (274)	74.1% (2108)	16.2% (462)	6.0% (170)	67.3% (1915)	26.7% (759)
Less than 5,000	8.8% (775)	82.4% (7218)	8.8% (767)	1.8% (162)	61.0% (5344)	37.1% (3254)	1.6% (139)	32.8% (2873)	65.6% (5748)	15.5% (1361)	69.6% (6098)	14.9% (1301)	9.8% (777)	73.9% (5845)	16.3% (1288)	6.2% (488)	67.5% (5336)	26.4% (2086)
χ^2^	*p* = .044	*p* = .929	*p* < .001	*p* = .152	*p* = .793	*p* = .100	*p* = .359	*p* < .001	*p* < .001	*p* < .001	*p* = .850	*p* = .432	*p* < .001	*p* = .317	*p* = .027	*p* < .001	*p* = .953	*p* = .001

*IPTP* Instrumental parentification toward parents, *EPTP* Emotional parentification toward parents, *SWR* Satisfaction with the role, *SI* Sense of injustice, *IPTS* Instrumental parentification toward siblings, *EPTS* Emotional parentification toward siblings; Family SES, 0 is the lowest rung

^*^limited sample: *n* = 40,659 adolescents with siblings

Within the overall sample, 9.3% (*n* = 4440) of the sample experienced no level of IPTP, 2.2% (*n* = 1047) experienced no level of EPTP, 1.5% (*n* = 711) experienced no level of SWR, and 14% (*n* = 6725) experienced no level of SI. Within the sample of adolescents with siblings, 11.4% (*n* = 4616) experienced no level of IPTS and 7.3% (*n* = 2959) experienced no level of EPTS. There were differences in the prevalence of no, low, and high parentification based on demographic variables.

#### Age Differences in Response Prevalence

In the late adolescent subsample (18–21), participants were more likely to exhibit high levels of IPTP and less likely to exhibit no level of IPTP as compared to those in early (12–14) or middle adolescence (15–17). Adolescents in middle adolescence (15–17 years old) were less likely to exhibit high levels of EPTP as compared to those in early and late adolescence. Adolescents in middle and late adolescence were more likely to exhibit no SWR or low levels of SWR as compared to those in early adolescence. In contrast, adolescents in early adolescence were more likely to exhibit high levels of SWR as compared to either middle or late adolescence, respectively. Adolescents in early adolescence (12–14 years old) were more likely to exhibit no SI and less likely to report high levels of SI as compared to adolescents in middle or late adolescence. For those adolescents with siblings, older adolescents were slightly more likely to exhibit high levels of IPTS as compared to younger adolescents. Additionally, older adolescents were less likely to exhibit no levels of EPTS as compared to younger adolescence.

#### Gender Differences in Response Prevalence

Significant gender differences were found based on the prevalence of response options for the six parentification subscale scores. Females were more likely to report no or low levels of IPTP as compared to males, who were more likely to report high levels of IPTP. In contrast, females were more likely to report high levels of EPTP as compared to males, who were more likely to report no or low levels of EPTP. Females were more likely to report low levels of SWR as compared to males who were more likely to report high levels of SWR. However, there were no gender differences with regards to no level of SWR. Females were more likely to report low levels of SI as compared to males. Males, in contrast, were more likely to report both no level and high levels of SI as compared to females. With regards to adolescents with siblings, there were no gender differences with regards to high levels of IPTS. However, males were more likely to report no level of IPTS, while females were more likely to report low levels of IPTS. Males were more likely to report either no level or low levels of EPTP as compared to females, who were more likely to report high levels of EPTP.

#### Family SES Differences in Response Prevalence

There were significant differences in the response category options for the parentification subscale scores based on “ladder rung” of the family SES. Adolescents indicating that they are in the highest and lowest SES categories were more likely to report either no level or a high level of both IPTP and SI as compared to those in the middle SES categories. In contrast, those indicating the middle SES categories were more likely to report low levels of IPTP and low levels of SI as compared to those in either the highest or lowest SES categories. The frequency of high values of both EPTP and SWR increased as the SES category reported by the adolescent increased. In addition, the frequency of no EPTP and no SWR decreased as the SES category increased, except for the highest SES category, which was similar to the lower SES categories in terms of frequency of no level of both EPTP and SWR. The level of both low EPTP and low SWR was the highest in the middle SES categories, with the frequencies of both low EPTP and SWR increasing as the adolescent either increased or decreased their SES category.

For those adolescents with siblings, frequencies of no IPTS are the highest for those in the lowest two reported SES categories, with significantly lower and a fairly equal distributions of no level of IPTS across the other SES categories. The highest frequency of high levels of IPTS were found for adolescents reporting the highest SES category and the third lowest SES category, with significantly lower values and a fairly equal distributions of high IPTS throughout the other SES categories. Those reporting the highest and lowest SES categories reported the lowest frequencies of low IPTS, with the other SES categories having significantly higher and approximately equal frequencies of low IPTS. Adolescents increased their likelihood of reporting high levels of EPTS as their reported SES category increased. Those indicating the highest and lowest SES categories had the lowest frequencies of low EPTS, with each of the other categories higher and approximately equal to each other in their frequencies of low EPTS. Those reporting the lowest SES categories had the highest likelihood of reporting no EPTS, with the likelihood decreasing as the reported SES category increased. The only exception to this increase is for those reporting the highest SES category, whose frequency of no EPTS was similar to the second to lowest SES category.

#### City Size Differences in Response Prevalence

There were significant differences in the response prevalence for five of the six parentification subscales based on the size of the city in which the adolescent lived, with no significant differences found for the EPTP subscale. Adolescents living in larger cities were less likely to report high levels of IPTP. Adolescents living in smaller cities were more likely to report high levels of SWR as compared to those living in larger cities, who were more likely to report low levels of SWR. Adolescents living in smaller cities were more likely to report no level of SI as compared to adolescents living in larger cities. For adolescents with siblings, those living in larger cities were more likely to report no level of both IPTS and EPTS and less likely to report high levels of both IPTS and EPTS as compared to adolescents living in smaller cities.

## Discussion

The findings provide the first prevalence data on parentification in a broad nationally representative sample of Polish adolescents. The most surprising result was the magnitude of emotional parentification toward parents. More than one-third of the total study sample declared a very high level of this type of parentification (35.9%). In comparison, a very high instrumental parentification toward parents was found in 7.2% of the total sample. Moreover, in case of parentification toward siblings, emotional parentification was more prevalent than instrumental parentification (25.2% vs. 15.5%, respectively). Those results are concerning because emotional parentification is identified as the most harmful type of parentification (Byng-Hall, 2008; McMahon & Luthar, 2007; Tompkins, 2007).

If more than one-third of Polish adolescents experience emotional boundary-crossing with their parents, it suggests that the source of this problem may be widespread. For example, the susceptibility to overshare with children, assigning them emotional roles, or being prone to enmeshment can be somehow related to something rooted in the Polish culture, or be an effect of the events that the generation of parents shared. The substantive role of power distance in the Polish families may provide some insight into the widely prevalent experience of emotional parentification in Polish adolescents. High power distance means that Polish families are strongly hierarchized (Hofstede et al., 2010). Therefore, even while feeling inconvenient, children and adolescents in Poland may feel the need to follow orders and tasks delegated by their parents. At the same time, Polish children may be less willing to openly discuss their feelings about being overburdened with family caregiving duties (see Ipsos Loyalty, 2014) because of generally strong commitment and restraint to social norms (Hosftede et al., 2010). Therefore, the rules such as showing respect to one’s parents, strong parental authority, and dealing with problems in a closed family system, may promote conditions for the parentification process (see Noivo, 1993; Schier, 2014).

Although there is a move toward Western democratic values such as family egalitarianism, traditionalism still is evident in Polish parenthood and families (Wejnert & Djumabaeva, 2005). For example, a study by Kosakowska-Berezecka et al. (2018) showed that in Poland domestic work is still perceived through the lens of gender. Traditional family models are gendered, characterized by high parental authority, and valuing the child’s obedience. With reference to parent–child relationships, they traditionally were mother-centered, due to the influence of the catholic patriarchal authority of the father, and the communist propaganda of the working mother model. This role model was not only working full-time, but also managing the family life, doing household chores, raising children, or taking care of elderly family members (Hryciuk & Korolczuk, 2012). Therefore, the traditional Polish parenting was expected to be based on strong emotional ties between ‘doing it all’ mother and a child, with loose or no ties between father and a child at the same time (Wejnert & Djumabaeva, 2005). Additionally, society’s perception of the archetypic strong, heroic, self-sacrificing mother (*Matka Polka*, i.e., the Polish Mother) is a symbolic figure present in the language, national identity, and popular culture, that puts high expectations and pressure on women. At the same time, this script reducing women to the family roles and motherhood only is anachronic in modern times. For example, due to the economic situation forcing both parents to provide for the family, new models of personal success, and women's rights movements (Hryciuk & Korolczuk, 2012).

However, even if outdated in modern times, those traditional family models shaped the parents of modern Polish adolescents. Those roles could have fostered high levels of closeness between mothers and their children what may make Polish families more prone to crossing the intergenerational boundaries. We think that the pressure of striving for perfection may paradoxically lead parents to a feeling of failure, low self-esteem, and satisfaction with the role, and in turn crossing the parent–child boundaries (e.g., looking for comfort and support in the child). Therefore, future studies on parentification should investigate if Polish mothers and fathers expose their children to different levels of parentification (comp. study from a German sample, Schier et al., 2015).

At the same time, the disproportion between overly engaging the children in emotional family caregiving, but less in instrumental roles, underlines how running the house and having the children ‘served’ is important for the Polish parents. Maybe the physical care and organizing the house chores are so important part of intergenerationally transmitted perception of parenthood that those types of chores are less eagerly delegated to the children?

Among the three age groups, high level of instrumental parentification toward parents and siblings was more prevalent in late adolescents (18–21 years old) than in early or middle adolescence. Also, high sense of injustice was the least prevalent among early adolescents and they were most likely to be highly satisfied with their family roles. Those results may suggest that assigning tasks and responsibilities in Polish culture, especially instrumental ones, may be based on taking into consideration the child’s age. Therefore, culture may be a potentially protective factor here (Jurkovic, 1997; Schier, 2014). On the other hand, prevalence rates of emotional parentification are mixed and harder to interpret. That may suggest parents who turn to their children to satisfy emotional needs may be doing it regardless of the child’s age.

The adolescent’s age has a large effect size only on the reference to the level of sense of injustice and satisfaction with the family role. The developmental stage does not seem to intervene with the levels of parent/siblings-focused types of parentification (low effect sizes). The older were the participants, the less they were satisfied with their family roles and declared more sense of injustice. This result may however be rooted in the developmental dynamics of intergenerational conflict adolescents and their parents experience while the adolescents are building their identity. However, the frequency of experiencing the highest level of injustice reaches 17.7% for late adolescents, 16.3% for middle adolescents, and 13.5% if early adolescents. All those young people who report feeling burdened and treated unfairly in their homes may be potential victims of severe neglect.

Parentification among Polish adolescents seems to be gendered. The prevalence of high instrumental parentification toward parents was substantially higher in boys than in girls, similar to findings evidenced by Lewandowska-Walter et al.’s (2017). Emotional parentification toward parents was more prevalent among girls. Boys were also more likely to report high sense of injustice or to be more satisfied with their roles in the families, similar to findings evidenced by Lewandowska-Walter et al. (2017) but the lack of this satisfaction was as prevalent among girls as it was among boys. Taking into consideration that emotional parentification is the most detrimental form of parentification, it is not surprising that boys were more satisfied with their roles. The adolescents declaring the highest sense of injustice could be those who experience emotional parentification, which in boys may be also accompanied by a sense of injustice – caused by breaking the conservative male gender role (e.g., East, 2010; Khafi et al., 2014; McMahon & Luthar, 2007).

However, the instrumental caregiving for the siblings was similarly prevalent among girls and boys, but having no instrumental parentification toward siblings was more frequent in boys. Additionally, girls scored higher in emotional parentification toward siblings and it was more frequent for boys not to experience it. One interpretation of these findings is that parentification in Polish adolescents is gendered due to the gendered perception of house and caregiving chores, similarly to the results of a study by Kosakowska-Berezecka et al. (2018).

The relations between the family SES and parentification dimensions varied and were conflicting and unexpected. For example, youth from the most financially privileged and the least privileged families presented the highest prevalence of instrumental parentification toward parents and the highest sense of injustice. On one hand, this result is in line with studies that show both low (e.g., Burton, 2007; Chee et al, 2014) and high family SES (see Carroll & Robinson, 2000; Winton, 2003) may be linked with parentification. On the other hand, the more financially advantaged the family was, the higher were the levels of satisfaction with the role (see Borchet, 2018), emotional parentification toward parents (see Winton, 2003), and emotional parentification toward siblings. Moreover, adolescents from high SES families reported a higher prevalence of emotional parentification toward parents than the general sample. This result is similar to Winton’s (2003) assertion that the specificity of parentification in high SES families may be emotional. It takes the form of children fulfilling parental expectations, being competitive in the social comparison, or replacing the spousal figure for their mothers while fathers are withdrawn from the family and focused on the career (Winton, 2003). Additionally, adolescents from both the richest and the poorest families reported the lowest frequencies of low instrumental parentification toward siblings, which was the most prevalent among middle-SES adolescents. Taken together, those conflicting results may suggest that (a) family SES may not be a linear predictor of parentification in Polish culture and (b) the type of parentification (i.e. emotional or instrumental) is important in the relation between family SES and parentification prevalence.

The results related to parentification dimensions and inhabited city size were mostly unexpected and seem to be different for every parentification type. Emotional parentification toward parents is the same prevalent regardless of the inhabited city size. It is another argument for the broad, generational source of this type of parentification in Polish culture.

Adolescents living in smaller cities were less likely to experience high level of sense of injustice and high levels of satisfaction with their role. That would suggest that this group is endangered with negative consequences of parentification. However, it is not that clear as youth from smaller cities seem to experience high instrumental parentification toward parents, and both instrumental and emotional parentification toward siblings more frequently than youth from bigger cities. Although the differences between big cities, middle-size cities, small cities, and villages in Poland are decreasing, they are still evident. The factors connected with the city size such as poverty, low wage, and unemployment (Główny Urząd Staystyczny [Statistics Poland], 2019) are in turn related to, for example, risk of depression, substance abuse, and risk of social exclusion (see Compton et al., 2014; Kerr et al., 2017). Living through such difficult circumstances may push children to help themselves and their families financially as well as give physical work that can help manage and visibly change the family’s everyday life. Moreover, the ecological context of the family life significantly impacts the adolescent's living conditions (e.g., proximity to the hospitals, psychological care, educational and cultural institutions, or transportation exclusion) what in turn may also affect the experience of parentification. For example, the child – family abuse victim living in a small town that experiences parentification may not have a place to seek psychological support or have trouble getting to the place where it is available (e.g., money for the ticket depends on the parents or there may be no bus or train driving through this town). Summing up, the environmental context in the parentification process can not be overestimated and requires further analysis.

## Limitations and Future Directions

The findings of this study have to be seen in the light of the study limitations. Design limitations of the study include cross-sectional assessment and the online data collection method. Moreover, the study lacked information about the participant’s ethnicity, and response options for gender were delimited to female and male. Importantly, the effect sizes for most of the differences are relatively small, indicating that the differences that we detected may lack practical significance. Given the size of the sample for our current study, it is not surprising that the analyses detected small differences. While we have controlled for error, and thus can attest that these differences are statistically significant, in some cases the significant differences may not be meaningful within the sample. On the other hand, given the range of response options (1–5), it is difficult to determine what may be a meaningful difference within the parentification scale. This note provides a limitation to our findings in that not all of the mean differences found above may be meaningful within adolescent parentification, though they are all significant.

Despite these limitations, the study provided the estimates of parentification dimensions prevalence among the population of Polish adolescents. Future studies on parentification among Polish as well as international adolescent samples should focus on the examination of the intersection of the sociodemographic factors shaping the parentification process in the family (e.g., family SES, divorce, number of children in the family, birth order). Cluster analysis could emerge patterns of coexistence between parentification risk and protective factors, as well as family characteristics. That knowledge could help to prepare comprehensive and culturally tailored intervention and prevention strategies to help improve Polish adolescents’ quality of life and facilitate their development. Also, the specific effects of mother- and father-focused parentification should receive the attention of the academic community, especially in Poland, due to its cultural relevance. Studies employing not only urban samples are much needed too.

These data have some potential intervention implications such as targeting samples such as older adolescents, girls, low and high SES families, and youth from smaller cities. These findings may help to raise awareness on the topic of parentification among parents, and various specialists working with children, adolescents, and families in Poland and abroad. Additionally, this study may lay the groundwork for follow-up studies of risk and protective factors, consequences, and early expressions of parentification in Polish adolescents as well as inspire future international research on parentification prevalence – what is a gap in the literature. Looking for more culture-specific factors that shape parentification in Poland is highly needed, as this phenomenon seems not to be best described by variables such as family SES. Moreover, we believe the described prevalence study would facilitate a broad international discussion on the context, antecedent, and outcomes of children and adolescents who experience parentification. Additionally, we underscore the need to expand the conversation on the often-reported emotional burden and how that outcome is perceived in diverse cultural contexts, communities, and families.

## Appendix

Study Sample by Voivodeship (*N* = 47,984)
